# Morphology and electrical characteristics of p-type ZnO microwires with zigzag rough surfaces induced by Sb doping†

**DOI:** 10.1039/c8ra07135g

**Published:** 2018-10-12

**Authors:** Linlin Shi, Luchao Du, Yingtian Xu, Liang Jin, He Zhang, Yan Li, Xiaohui Ma, Yonggang Zou, Dongxu Zhao

**Affiliations:** State Key Laboratory of High Power Semiconductor Laser, Changchun University of Science and Technology No. 7186 Wei-Xing Road Changchun 130022 People's Republic of China zouyg@cust.edu.cn +86-85583395 +86-85583395; State Key Laboratory of Luminescence and Applications, Changchun Institute of Optics, Fine Mechanics and Physics, Chinese Academy of Sciences No. 3888 Dongnanhu Road Changchun 130033 People's Republic of China; Institute of Atomic and Molecular Physics, Jilin University No. 2699 Qian-Jin Road Changchun 130012 People's Republic of China

## Abstract

Sb-doped p-type ZnO microwires with zigzag rough surfaces were synthesized by two zone chemical vapor deposition. The zigzag morphology characteristics analyzed by high resolution scanning electron microscopy and transmission electron microscopy show the existence of surface defects caused by Sb doping. The incorporation of Sb into a ZnO lattice induces lattice imperfection, which is the origin of the zigzag rough surface. Photoluminescence and electrical properties of the obtained Sb-doped ZnO microwires were determined. The crossed structure microwire-based p–n homojunction device was fabricated by applying as-synthesized Sb-doped p-type ZnO microwires and undoped n-type ZnO microwires. The doped microwires demonstrate reproducible p-type conduction and enhanced rectifying behavior with increasing Sb doping concentration. The results demonstrated that the optimizable optical and electrical characteristics, controlled by increasing the doping concentration, are reflected in the surface morphology changes which would be helpful for characterizing the doping effects in micro/nanoscale materials.

## Abstract 2

Sb-doped microwires which have a zigzag rough surface demonstrate p-type conduction and enhanced rectifying behavior with increasing Sb doping concentration.

## Graphical abstract

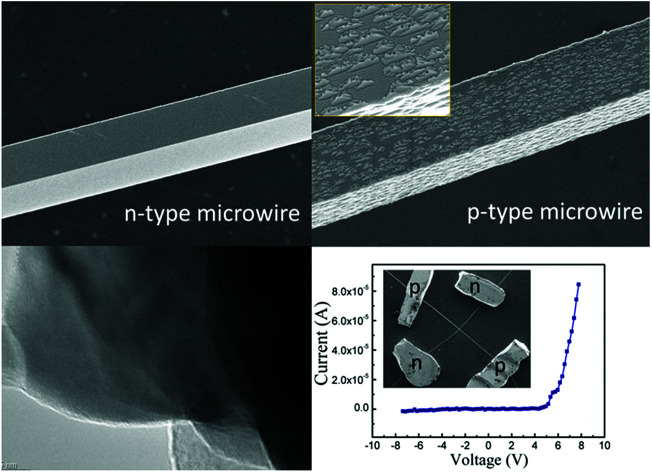

## Introduction

Single crystalline metal oxide semiconductor micro/nanomaterials have been utilized as building blocks in many photonic and electronic applications.^1–3^ Because of their superior properties, such as multi-functionality, high crystallinity, quantum size effects, high transparency, and stability in air, micro/nanomaterials can act as future electronic material alternatives to bulk materials. ZnO is one of the promising materials for the fabrication of optoelectronic devices operating in the blue and ultraviolet regions because of the wide direct band gap (3.37 eV) and large exciton binding energy (60 mV).^4,5^ p-Type doping in ZnO is essential for device applications to meet the increasing needs of present applications, and enormous efforts have been dedicated to the doping of ZnO with selective elements to fabricate homojunction devices, which have the advantages of high sensitivity, fast response speed, and high stability in various environments.^6,7^ However reports based on the homojunction devices of ZnO nanostructure are relatively rare; stable and reproducible p-type doping of ZnO is still a great challenge.^8–11^ Much effort has been made to obtain high quality p-type ZnO, it had been reported that Sb element with a large atom size is a good choice of ZnO dopant.^12–14^ Sb atom would substitute for a Zn atom and simultaneously produce two corresponding Zn vacancies, therefore Sb-doped ZnO shows strong p-type conduction based on first-principles calculation,^15^ hence large-mismatched Sb atoms are efficient p-type dopants for ZnO nanowires.^16–18^

Recently, various synthesis methods have been developed to synthesize micro/nanomaterials with controlling of their size and shape, including both vapor phase and solution phase synthesis, to enhance the multi-functionality of ZnO in different application fields.^19,20^ ZnO microwires with a novel square cross-section have promising prospect in the field of cavity quantum electrodynamics and the development of miniature optoelectronic devices.^21,22^ The electrical, optical, and magnetic properties of ZnO nanostructures can be drastically modified by impurity doping.^23,24^ Moreover, more complex ZnO structures can be developed when impurities are mixed into the source material. Some researchers reported that Sb in ZnO have an effect on the morphology of nanostructure.^25–27^ The introduction of Sb can alter the morphology of the ZnO nanomaterials, including crystal size, orientation, and aspect ratio, which also have important effects on their electrical and optical properties, gas sensitivity and catalytic properties.^28^ When Sb was introduced into ZnO, it has higher resistance and diffuse reflectivity than those of the undoped ZnO. But to our knowledge, there is no report concerning p-type conduction properties affected by morphology variation in Sb-doped ZnO.

Furthermore, micro/nanowires with 1D nanostructure have promising applications in developing novel devices. Novel devices constructed by two crossed micro/nanowire components to create efficient nanoscale p–n junction diodes have been previously configured as diodes successfully.^29,30^ The ability to fabricate micro/nanowire heterostructures should further increase the versatility of nanowire based electronic and photonic devices and also provide convenient subsequent assembly processes.^31,32^

In this study, ultralong Sb-doped ZnO single crystalline microwires were synthesized by vapor phase synthesis, the obtained Sb-doped ZnO microwire showed high crystalline in both zigzag surface area and inside area. P-type properties of ZnO microwires were examined by photoluminescence and electrical measurements, and the morphology and electrical properties changes with the concentration of Sb-doping. The crossed p–n homojunction was fabricated to identify p-type properties of zigzag by crossing a single Sb-doped ZnO microwire and a single undoped ZnO microwire.

## Results and discussion

### Morphologies and characterization

**Fig. 1:**
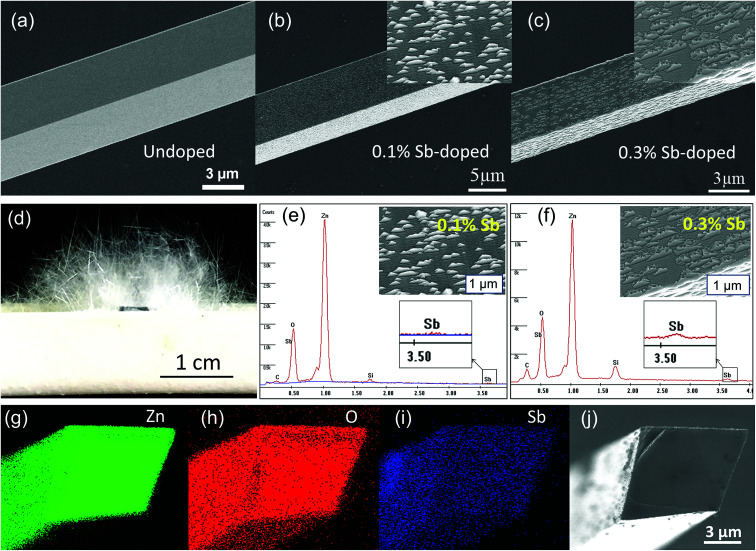
SEM images of the (a) undoped ZnO microwire shows a smooth surface. (b) 1% Sb-doped ZnO microwire shows a zigzag rough surface. (c) 3% Sb-doped ZnO microwire shows more obviously zigzag rough surface. Inset: the enlarged SEM images of Sb-doped ZnO microwires. (d) Optical image of the as-synthesized ultralong Sb-doped ZnO microwires. (e and f) EDX element analysis of 0.1% and 0.3% Sb-doped ZnO microwires, (g–j) the elemental mappings of Zn, O, Sb of the cross section of a Sb-doped ZnO microwire.

The as-synthesized ZnO microwire was analysed by Scanning Electron Microscope (SEM) to demonstrate the structural characters. The morphologies of both undoped and Sb-doped ultralong ZnO microwires with square cross-section are revealed in Fig. 1a–c. The surface morphology changes distinctly due to the incorporation of the Sb dopant, the microwire presents a zigzag rough surface compared to the smooth surface of the undoped ZnO microwire. Fig. 1d displays optical imaging of the as synthesized ultralong ZnO microwires on an alumina boat, the length can reach to about 10 millimeters. To determine the composition of Sb in ZnO microwires, we performed the Energy Dispersive X-ray spectroscopy (EDX) composition analysis taken in various regions to investigate Sb and Zn distribution. The morphology variation was investigated, it changes with the concentration of the Sb element in the microwire, Sb concentration was 0.1% and 0.3% in Sb-doped microwires within the measurement limit of EDX. The surface of zigzag roughness increases with the doping concentration, the details are shown from the inset enlarged SEM image in Fig. 1e and f. With the increase of doping concentration, triangle embossment became intensively and obviously in 0.3% Sb-doped microwire. To further confirm the existence of Sb-doping, the cross section element mappings of individual Sb-doped ZnO microwires using EDX are depicted in Fig. 1g–j. Zn, O and Sb are homogeneously and uniformly distributed in the microwires. The inset is the local magnified portion with the energy from about 4 keV to 6 keV, indicating the existence of Sb in the doped material.

**Fig. 2:**
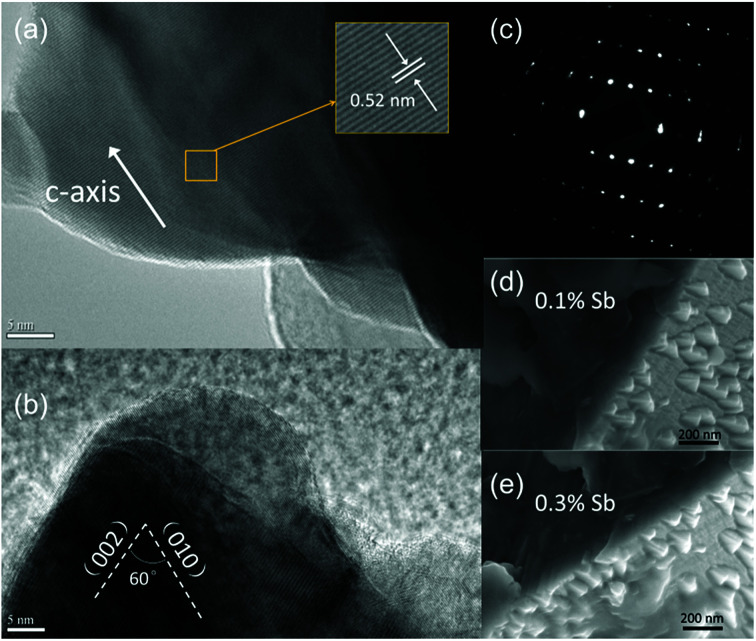
(a and b) HRTEM image taken from the zigzag area of ZnO microwire shows high quality crystallite. (c) The corresponding SAED pattern. (d and e) SEM images amplification of the edges of Sb-doped ZnO microwire.

To understand the origin of the novel zigzag surfaces in the microwires, the corresponding magnification of triangle edges of the Sb-doped ZnO microwire were further determined by high-resolution TEM and SEM analysis. The results are recorded in Fig. 2. Detailed information can be obtained from the clear lattice structure, indicate good crystal quality. The *c*-axis growth direction with *d*-spacing of 0.52 nm was indicated by adjacent lattice spacing, it can be indexed into [0001] growth orientation, which is the favoured growth direction of ZnO microwire. The angle measurement of edges with respect to the [002] direction revealed an angle of ∼60° between the two planar defects,^25^ which is consistent with the angle relationship between the (010) and (002) planes in the SAED pattern.^33^ The corresponding selected area electron diffraction (SAED) pattern in Fig. 2c reveals that the growth orientations is along [0001]. The internal structures of the Sb-doped ZnO microwires were further characterized by enlarged SEM characterizations of triangle zone of microwire surface which are shown in Fig. 2d and e. The enlarged images of the edges of ZnO microwire display a smooth transition of the edges of ZnO display a smooth transition between the zigzag surface and internal microwire, both in 0.1% Sb-doped (Fig. 2d) and 0.3% Sb-doped (Fig. 2e) microwire. The above results reveal that Sb-doped ZnO microwires were highly crystallized with zigzag surface.

The origination of the zigzag rough surface in ZnO microwires could be attributed to the introduction of Sb dopants. Sb element is responsible for the formation of the single-side zigzag boundaries in the nanowires, the radius of Sb^3+^ (0.078 nm) is much larger than that of Zn^2+^ (0.074 nm), the introduction of Sb^3+^ ions should cause a large structural strain. Since the radius of Sb^3+^ (0.78 Å) is much larger than that of Zn^2+^ (0.74 Å), the introduction of Sb atoms would cause a large structural strain, the release of the structural strain could result in the formation of the planar defects.^34^ When Sb in ZnO, the surface energy of (001) planes is reduced, crystal growth along the (001) orientation is suppressed, and then the morphology of the ZnO micro/nanowires can be changed. ZnO microwires usually grow along the *c* axis, and the side surfaces are {0110} and/or {2110} because of the lower energy surfaces compared to (0001).^25^ The solutes prefer to segregate from the host and occupy the surface of the nanostructures, result in a decreased surface energy.^26^ Hence we propose that Sb is responsible for the zigzag surface and zigzag shaped microwires are surrounded by (0002), (0110) and (2110) as the surface.^27^

### Growth mechanism

**Fig. 3:**
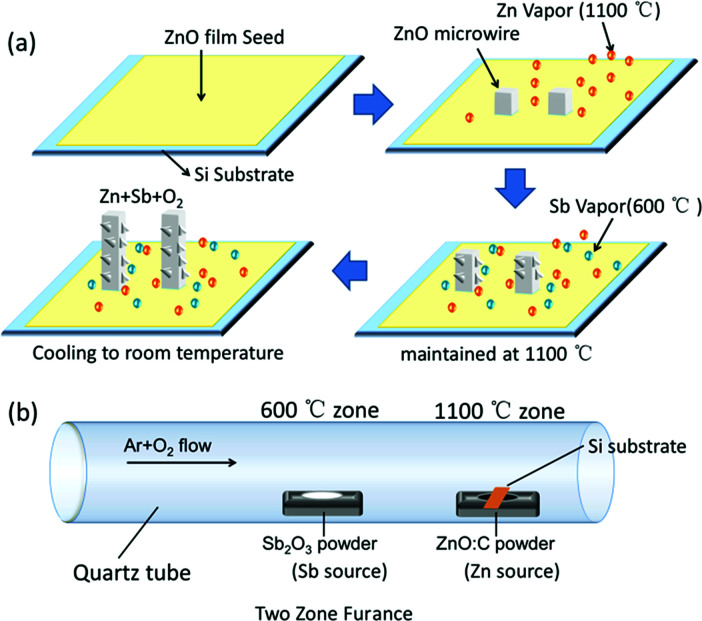
(a) Schematic diagram of the growth process of the Sb-doped ZnO microwire, (b) schematic diagram of the two zone growth condition.

In our experiment, the doping procedure performed under the two zone *in situ* synthesis condition, the synthesis condition is shown in Fig. 3. The Sb source served as the dopant source material could vaporize at the same speed with Zn source. When the substrate was heated to about 1100 °C, oxygen was introduced for about 2 min to keep the synthesis under the oxygen-rich conditions which would suppress the formation of Sb_O_. The rough surface morphologies become obviously with increasing the O_2_ flow, which is shown in Fig. S1.† Based on the above experimental process and relevant analyses, we proposed the growth mechanism of the doping procedure of *in situ* doping of Sb-doped ZnO microwire, the schematic diagram is shown in Fig. 3a. The reaction temperature of Sb source (Sb_2_O_3_ powder) is about 600 °C, which is much lower than the synthesis temperature of ZnO. By separating two heating zones, Zn and Sb vapor was generated simultaneously to the growth substrate with the temperature increasing, deposited on the growth substrate to form droplets. As the O_2_ gas was introduced into the furnace at 1100 °C, when the ZnO in the droplets reach a saturated concentration, Sb-doped crystalline ZnO begin to grow from the droplets. The initially formed Sb vapor from the source can provide the necessary feeding dopant material for the microwire growth. It suggests that Sb vapor is diffused full of the whole growth process, the surface zigzag morphology affected by the inside defect could contribute to the introduction of Sb atoms in the ZnO crystal growth process.^24^

### Optical properties

**Fig. 4:**
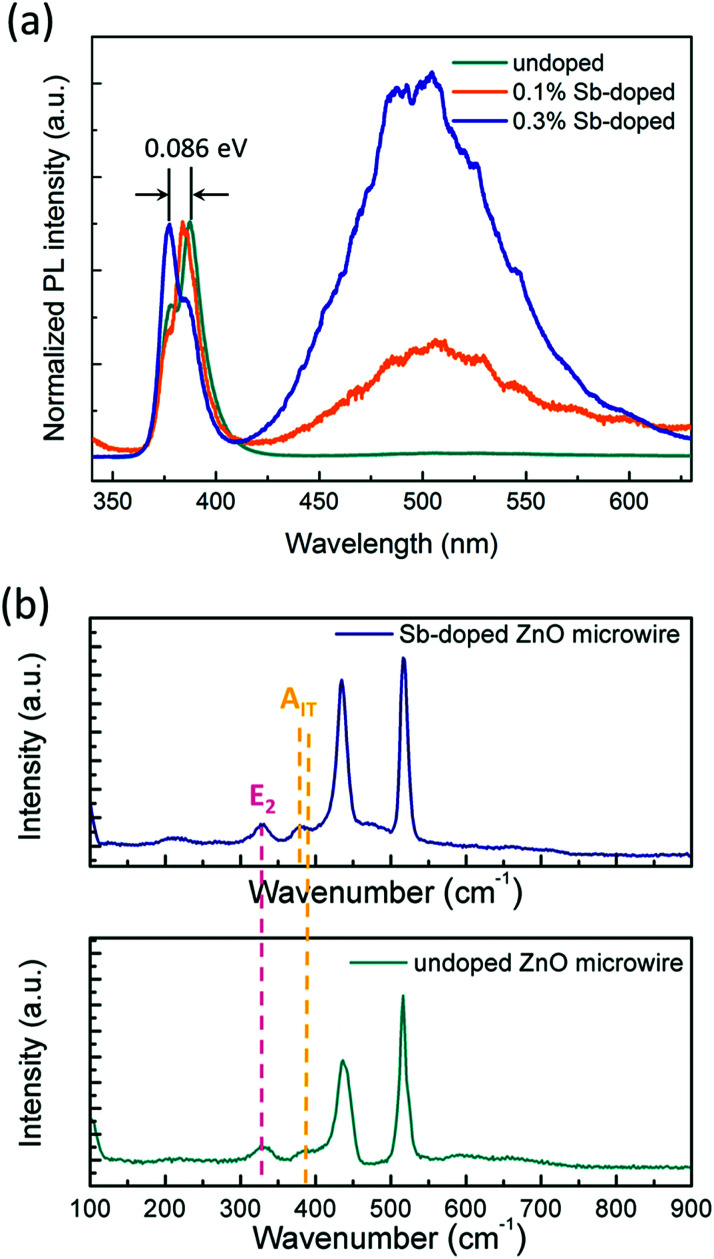
(a) Normalized photoluminescence of Sb-doped and undoped ZnO microwires at room temperature, (b) Raman spectra of Sb-doped and undoped ZnO microwires.

To investigate optical properties of the ZnO microwires, photoluminescence (PL) spectra were measured at room temperature using the 325 nm line of a He–Cd laser as an excitation source, the results are shown in Fig. 4a. As can be seen from the PL spectra, the near-band edge (NBE) emission centered at 382 nm can be mainly observed in both undoped and Sb-doped ZnO microwires, there are two emission peaks in Sb-doped ZnO microwires with an ultraviolet (UV) emission related to the NBE attributed to the exciton recombination of ZnO, and a broad green emission band related to defects at centered at 500 nm in both 0.1% and 0.3% Sb-doped microwires. The broad visible emission can be identified as deep level defects which usually caused by impurities and structural defects.^35^ According to the normalized PL spectrum in Fig. 4a, the intensity ratios of the visible emission to the UV emission of 0.3% Sb-doped microwire is larger than 0.1% Sb-doped microwire, the increasing defect emission peak could be result from the introducing of increased impurity atoms, indicates the decreased crystallinity of the zigzag ZnO nanostructure and effective Sb defects inside. Compared with the undoped ZnO microwire, the intrinsic emission of 0.3% doped microwire is at 377.37 nm (3.26 eV), with a marked deviation of 0.085 eV from the emission (387.65 nm, 3.19 eV) of undoped ZnO. For 0.1% Sb-doped microwire (384.04 nm, 3.23 eV), the deviation is 0.04 eV, which is smaller than the 0.3% Sb-doped microwire. This is probably due to the formation of acceptor levels by Sb doping. With the doping concentration increases, the intensity of defect emission peak increases and the zigzag rough surfaces become obvious.

The microstructure characterization and the optical property of the microwires were also investigated by the Raman scattering to further confirm the incorporation of Sb atoms into the ZnO microwire lattices. As shown in Fig. 4b, the peak centered at 436 cm^−1^ is usually attributed to the E_2_ modes of the undoped ZnO single crystal, and this peak shifts toward lower frequency to 434 cm^−1^ when doping with Sb, which is caused by the formation of the Sb_Zn_–2V_Zn_ low formation energy complex acceptor in Sb-doped ZnO microwires, the defect can be identified as represented the shallow acceptor defect levels.^34,36^ The peak at 380 cm^−1^ corresponds to the A_1T_ modes in ZnO is depressed by Sb doping. Since the oxygen defect or the dopant atom directly affects the A_1T_ modes, indicates that Sb dopant atoms would be responsible for the depression in 380 cm^−1^.^37^ Consequently, PL and Raman spectra characteristics reveal that Sb was incorporated into ZnO crystal lattices and the zigzag morphology have an impact on the emission according to Sb doping.

**Fig. 5:**
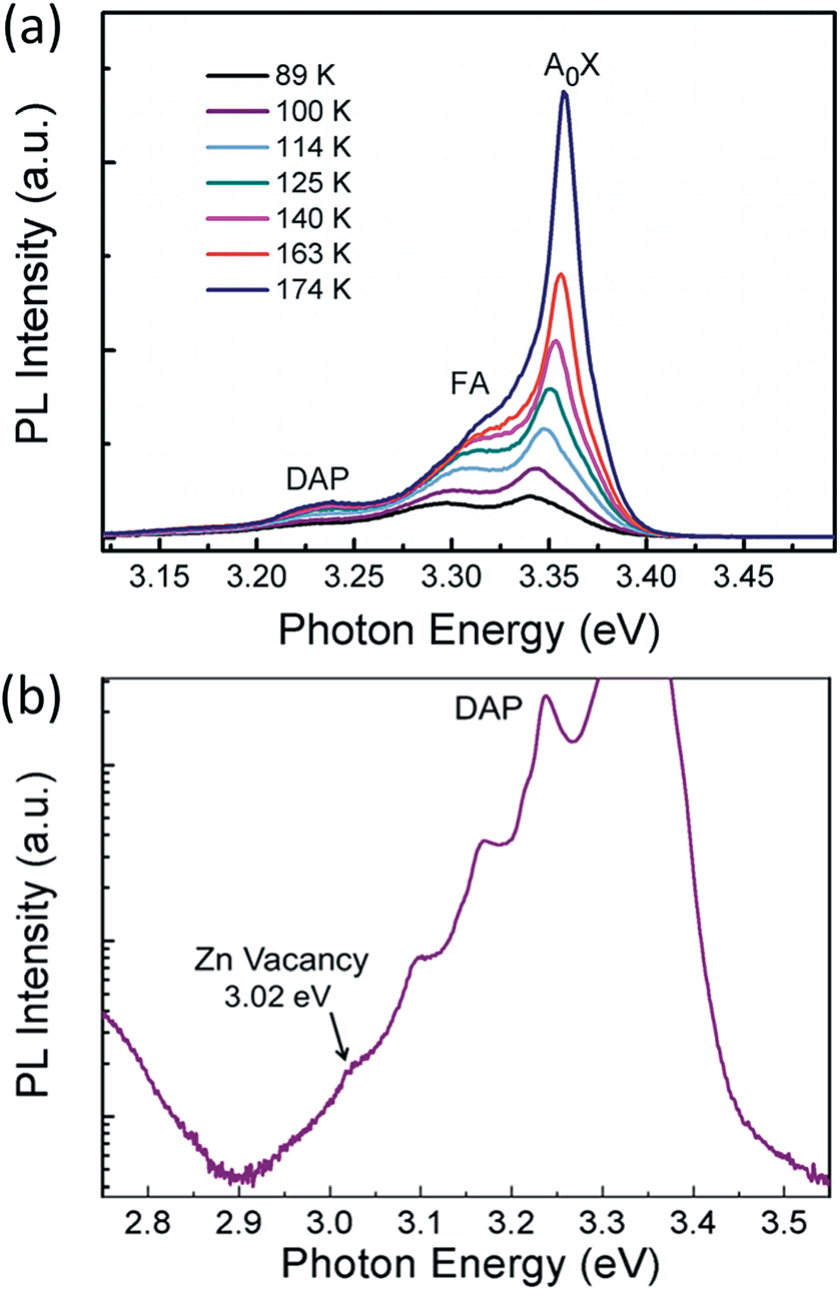
(a) Temperature dependent PL spectra of Sb-doped ZnO microwires. (b) Low temperature PL spectrum at 85 K in log scale, indicate that the Sb dopant has been incorporated into ZnO and introduced acceptor levels.

The p-type behaviour of the nanowires can also be confirmed optically by low-temperature photoluminescence (PL). The low-temperature (at 85 K) PL spectra of Sb-doped ZnO microwires were shown in Fig. 5a. The Sb-doped ZnO microwire spectrum shows a dominated emission peak centered at 3.354 eV, which is associated with the neutral-acceptor-bound exciton (A^0^X) recombination.^38^ The emission peaks located at 3.296 eV and 3.226 eV are ascribed to the free electron to acceptor (FA) and donor–acceptor-pair (DAP) transitions, respectively.^39^ The PL analysis suggests that the Sb-doped ZnO has shallow acceptor induced energy levels, which result in the p-type behavior of the microwires. The acceptor binding energy of EA in ZnO can be estimated by the following formula:1
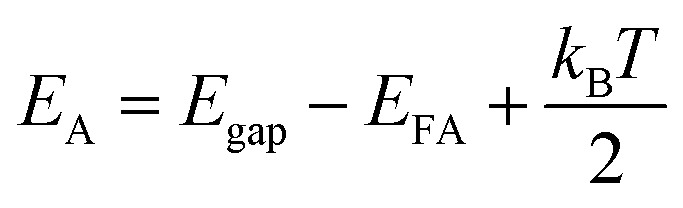
where *E*_FA_ is a free electron to the FA, *k*_B_ is the Boltzman constant, and *T* is the temperature. According to the equation, the acceptor binding energy of *E*_A_ can be calculated to be about 116 meV with the intrinsic band gap of *E*_gap_ = 3.41 eV, which is similar to the reported results for p-type ZnO nanowires.^13^ To further understand this emission, we investigated PL spectrum in log scale showed in Fig. 5b, the peak located at 3.02 eV is related to Zn vacancies (V_Zn_).^40^ Owing to the substituting of Zn atom by Sb dopant, the observation of evident Zn vacancy-related emission at 3.02 eV indicates the successfully doping of Sb. It is observed that Sb doping can result in the formation of Zn vacancies. The above results indicate that the incorporation of Sb atom could act as a suitable p-type dopant.

### Electrical properties

**Fig. 6:**
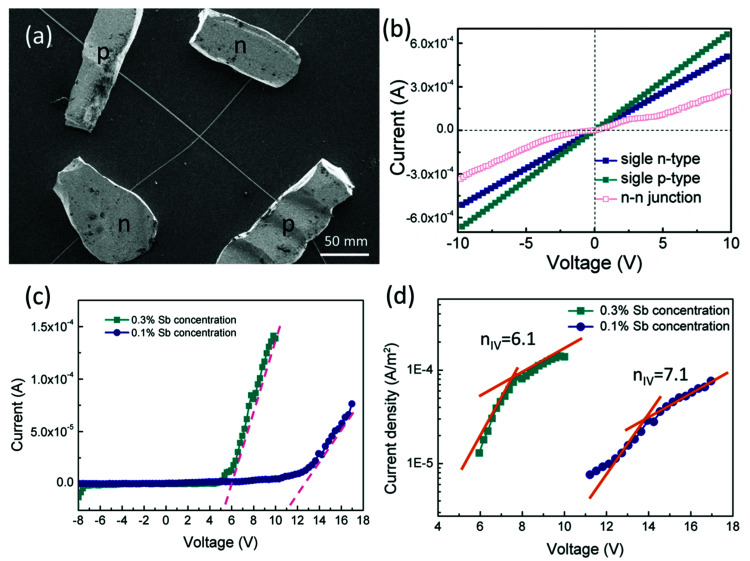
(a) SEM image of microwire based crossed p–n homojunction. (b) *I*–*V* characteristics of individual n-type and p-type ZnO microwires and crossed two single n-type ZnO microwires with n–n junction, (c and d) *I*–*V* characteristics of the crossed ZnO microwire p–n homojunction with 0.1% Sb concentration (c) and 0.3% Sb concentration (d).

To confirm p-type conductance behaviour, a microwire-based p–n homojunction were fabricated with a single Sb doped p-type ZnO microwire and a single undoped n-type ZnO microwire, microwires were placed overlapping with each other on the glass substrate to form a crossed structure, fixed with indium electrodes at each end to create a crossed architecture. The crossed architecture is a clear example since the key device properties are defined by assembly of the two microwire components, and have the advantage of easy fabrication.^41,42^ The SEM image of the crossed heterojunction is shown in Fig. 6. Two metal–semiconductor (MS) contacts were used for two electrodes, MS contacts have been found to have dominative influence on the transport behavior of 1D nanomaterials, so the individual microwires with MS contacts were tested, the results are shown in Fig. 6b. Transport data recorded on the individual p-type and n-type ZnO microwires both exhibit linear behaviors, indicate that individual microwires form an ohmic contact with the electrodes. Current–voltage (*I*–*V*) data for the crossed p–n junction (Fig. 6c and d) shows well-defined current rectification. Transport measurement carried out on the n–n junction by crossing two n-type ZnO microwires shows nearly linear behaviour, which indicates that the interface between individual n-type microwires does not produce a nonlinear curve. Consequently, the rectification can be definitely attributed to the crossed p–n homojunction. The p–n homojunction diode with a turn-on voltage of approximately 10 V in 1% Sb-doped microwire and 5 V in 3% Sb-doped microwire, which showed enhanced rectifying behaviour by increasing Sb doping concentration. The crossed p–n homojunction exhibits a rectifying behavior with a lower turn-on voltage tuned by increasing Sb dopant concentration is resulted from the Sb inducement. The initial turn-on voltage is approximate to the bandgap of ZnO, corresponds to injection of electrons into the p-ZnO from the n-ZnO. According to the IV characteristics of the real diodes,^43^2
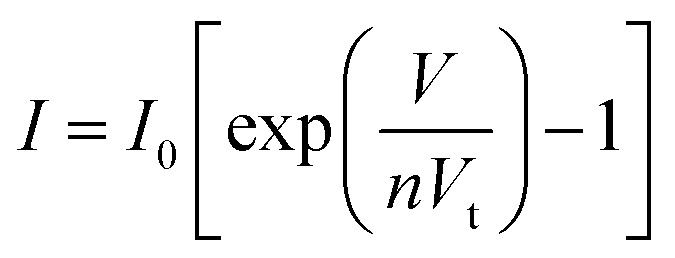
3
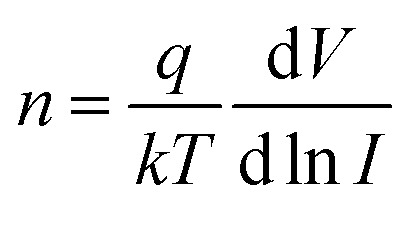
where the factor *I*_0_ is the reverse saturation current, *V* is the voltage at the junction, *V*_i_ = *kT*/*q* is the thermal voltage, *k* is the Boltzmann constant, *T* is the absolute temperature, and *n* is the junction ideal factor, which is determined from the slope of the straight line region of the forward bias log *I*–*V* characteristics.

According to the Sah–Noyce–Shockley theory, the value of the ideality factor in a p–n junction is about 1.0–2.0 at low or high voltage. However, the value of *n* for the ZnO p–n homojunction is higher than the ideal diode according to the equation and Fig. 6, this may result from the presence of nonlinear MS contact in the system and in the junction area.^44^ The basal diode can be modeled by a series of resistances in a dissimilar bias voltage range. The substitution of Sb into ZnO contributes acceptor characteristics, the electrons in the outer shell of Sb should be taken into account, the outer shell d states generally appear near the Fermi level. The bonding states have a higher energy level and are very close to the conduction band, and the electrons in this state have a higher probability of jumping to the conduction band, resulting in the increase of conductivity with the increase of Sb concentration in ZnO, which cause the increase of the turn-on voltage of p–n junction semiconductor diode.

## Conclusion

Sb-doped p-type ZnO microwires were synthesized by a CVD method, the morphology changed distinctly due to Sb incorporation. P-type properties of ZnO microwires were examined by photoluminescence and electrical measurements. Low-temperature PL and Raman spectra measurements showed that shallow acceptor was formed in the Sb-doped ZnO microwire. Fine rectification character was obtained on the crossed ZnO microwire p–n homojunction indicating the p-type conductivity of the Sb-doped ZnO microwire. The morphology and electrical properties changes with Sb-doping. The doped microwires demonstrate reproducible p-type conduction and enhanced rectifying behavior with increasing Sb doping concentration. The zigzag shape of ZnO microwires after introducing dopant could be a reflection of effective doping, the capability of testing p-type conduction characteristics and the understanding of emission behaviour of Sb-doped ZnO microwires have promising future applications of ZnO micro/nanowire based electronic and optoelectronic devices.

## Experimental

### Synthesis of ZnO microwire

Sb-doped ZnO microwires were grown by a catalyst-free chemical vapor deposition (CVD) method with a two-zone tube furnace. ZnO and graphite powders were thoroughly mixed with a weight ratio of 1 : 1, then the mixture was put into an alumina boat to act as the reactant source material. The Sb_2_O_3_ powders which served as the dopant source material were loaded into another boat at the upstream to ensure that Sb_2_O_3_ and ZnO powders could vaporize at the same speed. A 100 nm ZnO film was deposited on the Si substrate by the sputtering method, and then the substrate was loaded above the ZnO powders. During the synthesis progress, the two sources were loaded into the furnace at different temperature zones with a constant flow of 100 sccm argon (99.99%) as the carrier gas. The reactant ZnO source was heated to 1100 °C and the Sb_2_O_3_ dopant source was heated to 600 °C, simultaneously. When the ZnO source was heated to about 1100 °C, oxygen was introduced with a 20 sccm flow rate for about 2 min to keep the synthesis under the oxygen-rich conditions which would suppress the formation of V_O_ and Sb_O_. After maintaining the source temperature for 40 min, the furnace was turned off and cooled down to room temperature. The synthesis of the undoped ZnO microwires follows the same procedure without the Sb dopant source.

### Measurements

The morphology of the ZnO microwires was characterized by a field-emission scanning electron microscope (FE-SEM) (model: Hitachi S-4800) equipped with an X-ray energy dispersive spectrometer (EDX). The selected area electron diffraction (SAED) patterns were obtained on a JEOL JEM-2100F. Photoluminescence (PL) measurement was carried out with a JY-630 micro-Raman spectrometer employing the 325 nm line of a He–Cd laser as the excitation source, and the room temperature Raman spectra were recorded using the same micro-Raman spectrometer with a 488 nm excitation source. The current–voltage characteristics of the crossed p–n homojunction device were measured using a Keithley 2611A measurement system.

## Conflicts of interest

There are no conflicts to declare.

## Supplementary Material

RA-008-C8RA07135G-s001
