# Supplementary material for: Morphology and electrical characteristics of p-type ZnO microwires with zigzag rough surfaces induced by Sb doping
Source: RSC Adv. 2018 Oct 12;8(61):35023–30. doi: 10.1039/c8ra07135g (PMC9087441; doi:10.1039/c8ra07135g)
Supplement: RA-008-C8RA07135G-s001 [file RA-008-C8RA07135G-s001.pdf]

Electronic Supplementary Material (ESI) for RSC Advances.
This journal is © The Royal Society of Chemistry 2018

Morphology and electrical characters of p-type ZnO microwires with zigzag rough surfaces induced by Sb doping

According to the above discussion, the rough surface resulted from the existence of defect is effected by the growth atmosphere condition. It is reported that, the effective doping must avoid the V_O defect formation, which is a donor defect, not an acceptor defect lead to p-type conduction. Based on the first principles analysis, the effective doping needs to apply an oxygen-rich ambient, either during growth or during postgrowth thermal treatment.[1] Hence we introduced O_2 during the doping process. The morphology changes with the O_2 flow because oxygen atmosphere is a critical factor of successfully doping by suppressing the formation of V_O defect, which is disadvantage for the formation of p-type ZnO. The SEM of different is showed in Figure S1, it is suggested that the morphology have small differences under the same O_2 flow. With increasing the flow rate of O_2 increases, the rough surfaces become obviously, the morphology changes distinctly with the O_2 flow exceeding 30 sccm, but the concentration of Sb remains at 0.3% from EDS measurement

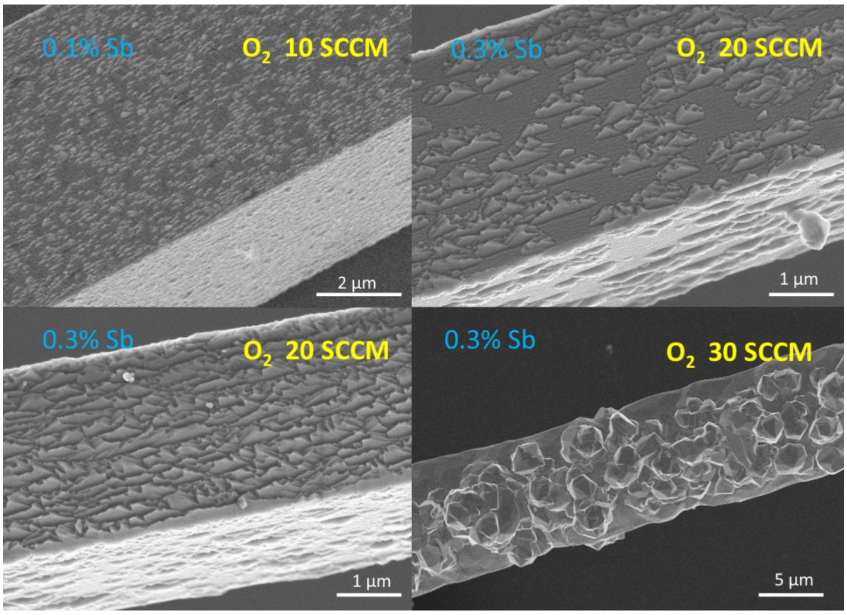

Figure S1. SEM pictures of the morphology controlled by O_2 flow.

Reference

[1] S. Limpijumnong, S. B. Zhang, S. H. Wei and C. H. Park, Doping by large size mismatched impurities the microscopic origin of arsenic or antimony doped p type zinc oxide, *Phys. Rev. Lett.*, 2004, **92**, 155504.
